# University of the Pacific

**Published:** 1859-11

**Authors:** 


UNIVERSITY OF THE PACIFIC.—Under this name, a name ominous we trust of its future harmony and prosperity, has recently been established, at San Francisco, the first medical school in California. The faculty consists of young, able, and efficient men, filled with zeal for the honor and advancement of medicine in our far-off sister State. We wish them all a happy and a prosperous career, and hope that the day is not distant when they may be able to build up an institution that shall be alike creditable to them and honorable to our beloved Union. Several of the members of the faculty of this new school have already earned an enviable reputation.

